# Towards an Algorithm-Based Tailored Treatment of Acute Neonatal Hyperammonemia

**DOI:** 10.3390/toxins13070484

**Published:** 2021-07-13

**Authors:** Sunny Eloot, Jonathan De Rudder, Patrick Verloo, Evelyn Dhont, Ann Raes, Wim Van Biesen, Evelien Snauwaert

**Affiliations:** 1 Department of Nephrology, Ghent University Hospital, 9000 Ghent, Belgium; jonthan.derudder@uzgent.be (J.D.R.); wim.vanbiesen@ugent.be (W.V.B.); 2 Department of Pediatric Metabolic Disease, Ghent University Hospital, 9000 Ghent, Belgium; patrick.verloo@ugent.be; 3 Department of Pediatric Intensive Care, Ghent University Hospital, 9000 Ghent, Belgium; evelyn.dhont@uzgent.be; 4 Department of Pediatric Nephrology, Ghent University Hospital, 9000 Ghent, Belgium; ann.raes@ugent.be (A.R.); Evelien.Snauwaert@UZGent.Be (E.S.)

**Keywords:** hyperammonemia, inborn errors of metabolism, hemodialysis, infant

## Abstract

Acute neonatal hyperammonemia is associated with poor neurological outcomes and high mortality. We developed, based on kinetic modeling, a user-friendly and widely applicable algorithm to tailor the treatment of acute neonatal hyperammonemia. A single compartmental model was calibrated assuming a distribution volume equal to the patient’s total body water (V), as calculated using Wells’ formula, and dialyzer clearance as derived from the measured ammonia time–concentration curves during 11 dialysis sessions in four patients (3.2 ± 0.4 kg). Based on these kinetic simulations, dialysis protocols could be derived for clinical use with different body weights, start concentrations, dialysis machines/dialyzers and dialysis settings (e.g., blood flow Q_B_). By a single measurement of ammonia concentration at the dialyzer inlet and outlet, dialyzer clearance (K) can be calculated as K = Q_B_∙[(C_inlet_ − C_outlet_)/C_inlet_]. The time (T) needed to decrease the ammonia concentration from a predialysis start concentration C_start_ to a desired target concentration C_target_ is then equal to T = (−V/K)∙LN(C_target_/C_start_). By implementing these formulae in a simple spreadsheet, medical staff can draw an institution-specific flowchart for patient-tailored treatment of hyperammonemia.

## 1. Introduction

Acute hyperammonemia is a rare but life-threatening condition in the neonatal period, most commonly caused by inborn errors of metabolism (IEM) such as urea cycle disorders [1,2] and organic acidemias [3,4]. A partial or complete inactivity of enzymes responsible for eliminating nitrogenous waste products results in acute hyperammonemic episodes [1,2,5,6]. During these events, ammonia diffuses into the brain where it exerts severe brain toxicity, resulting in severe neurological sequelae or even death [1,5,6,7]. Brain toxicity and neurological outcome is strongly related to the duration and severity of the hyperammonemia. Children’s IQ 12 months after recovery is inversely related to the duration of the hyperammonemic coma [8,9]. Therefore, early diagnosis and adequate management of hyperammonemia are considered key to improving the neurological outcome of affected neonates [1,10,11,12,13,14,15].

In neonates with inadequate clinical response to nitrogen-scavenging agents or with high ammonia levels (>500 µmol/L), renal replacement therapy to rapidly remove ammonium is recommended. Based on published case reports and retrospective studies in children from 1 day to 7 years old, the PCRRT (Pediatric Continuous Renal Replacement Therapy) workgroup recently published a Consensus Statement with guidelines for non-kidney replacement therapy (NKRT) and kidney replacement therapy (KRT) for hyperammonemia in pediatric patients [16]. When available, continuous veno-venous hemodialysis (CVVHD) or hemodialysis (HD) are recommended as the first-line treatment of acute hyperammonemia, with consideration of high-dose CVVHD or HD as initial therapy in patients with high serum ammonia levels (>1000 µmol/L), and severe and/or rapidly deteriorating neurological status [16].

Due to the limited number of cases on one hand, and the variety in timing of KRT initiation, KRT modality and prescription on the other hand, the proposed PCRRT consensus guideline is only supported by poor evidence and offers only limited practical recommendations. Hence, the guideline cannot simply be translated to any institution, dialysis prescription and setup. In order to come to more evidence-based guidelines in the choice and prescription of the KRT, a complete insight in ammonium transport and elimination in neonates with acute hyperammonemia in different dialysis modalities and setups is needed. Herewith, it is important to map the full-scale situation of the dialyzed patient. 

Therefore, a kinetic patient–dialyzer model has been derived for acute neonatal hyperammonemia based on our experience with infants on both the CarpeDiem^TM^ (Cardio-Renal Pediatric Dialysis Emergency Machine) (Medtronic, Minneapolis, MN, USA) [17,18] and 4008 (Fresenius Medical Care, Bad Homburg, Germany) dialysis machines. The model algorithm is provided to allow medical staff to derive institution- and patient-specific dialysis prescription once dialyzer clearances are known, and ensure a fast and successful decline in ammonia level.

## 2. Results

Four neonates (2.96–3.86 kg; three males; 3–5 days of age) with severe hyperammonemic coma were included, and were initiated on hemodialysis (HD) within 90 min (range 57–90 min) after arrival at the pediatric intensive care unit in our center (Table 1). Ammonia levels at HD initiation varied between 709 and 3017 µmol/L. Two neonates were already initiated on nitrogen-scavenging agents in the referring hospital (1 h 23 to 13 h 05 prior to hemodialysis initiation), while the other two patients were started on adequate nutritional and medical management including nitrogen-scavenging agents upon their arrival at our center.

Vascular access was in all patients obtained through 6.5Fr double lumen jugularis central venous catheters (Joline, Hechingen, Germany). The four patients underwent 11 HD sessions in total for which sufficient data were available for the kinetic analysis: i.e., five with the 4008 dialysis machine with FXPaed dialyzer, and six with the CarpeDiem^TM^ dialysis machine (two of them with 0.15 m^2^ and four with 0.25 m^2^ dialyzer) (Table 1). All patients were started on a 4008 dialysis machine with FXPaed dialyzer, and the dialysis circuit was primed with a solution of 50% Packed Cells and 50% albumin solution 5% (PC-5% Alb) (Alburex^®^, CSL Behring GmbH, Marburg, Germany). In the subsequent sessions, either 4008/FXPaed or CarpeDiem^TM^ 0.15 and 0.25 m^2^ were used and circuit priming was achieved either with PC-5% Alb or saline (Table 1).

Blood flows (Q_B_) were 30–35 (4008/FXPaed), 22–35 (CarpeDiem^TM^ 0.15 m^2^) and 30–34 mL/min (CarpeDiem^TM^ 0.25 m^2^), while dialysate flow was 300 (4008/FXPaed) and 10 mL/min (CarpeDiem^TM^). For all sessions, heparin was administered as anticoagulation.

**Figure 1:**
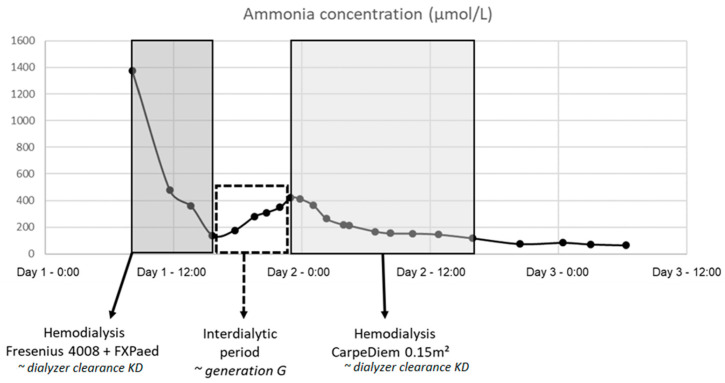
Ammonia concentration curve in patient 1: dialysis with 4008/FXPaed on day 1 and with CarpeDiem/0.15m^2^ on day 2.

Figure 1 shows the ammonia concentration curve in patient 1 (male, 3 days old, 3.15 kg, 48 cm), including dialysis with 4008/FXPaed on dialysis day 1 and with CarpeDiem/0.15 m^2^ on day 2. Overall dialyzer extraction ratios were found to be 45 ± 6% with 4008/FXPaed dialysis, and 13 ± 3% and 15 ± 4% with the CarpeDiem/0.15 m^2^ and CarpeDiem/0.25 m^2^ dialysis setup. Ammonia generation in the patients was 0.48 ± 0.32 µmol/min, with no observed impact on the calculation of dialyzer clearance and extraction ratio.

**Figure 2:**
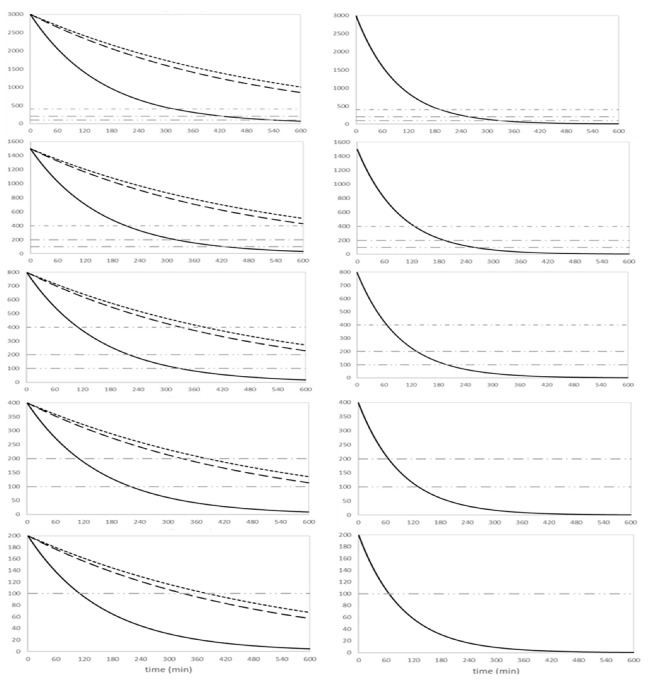
Time–ammonia concentration curves of the simulations in a 3 kg male infant, for different start concentrations and using different dialysis setups with a blood flow of 30 (left panels) and 50 mL/min (right panels). Dialysis setup 4008/FXPaed (full line), CarpeDiem/0.15 m^2^ (dotted line) and CarpeDiem/0.25 m^2^ (dashed line). Q_B_50 can only be executed with the 4008/FXPaed setup.

Based on the derived kinetic model, intradialytic simulations over 10 h were completed for a male infant of 3 kg and 50 cm, and for different dialysis start concentrations (i.e., 3000, 1500, 800, 400 and 200 µmol/L). The time–ammonia concentration curves are shown in Figure 2 for a blood flow of 30 (left panels) and 50 mL/min (right panels).

The time interval needed for start ammonia concentrations to reach the different thresholds (i.e., 400, 200 and 100 µmol/L) is shown in Table 2 and Table 3 for an infant of 3 and 4 kg, respectively. Using the 4008/FXPaed, the time to decrease from start concentrations of 3000 to <400 µmol/L was 322 min for a Q_B_ of 30 mL/min and 193 min for a Q_B_ of 50 mL/min, while it was 211 and 126 min for a start concentration of 1500 µmol/L, and 111 and 66 min for a start concentration of 800 µmol/L. In general, for ammonia start concentrations >800 µmol/L in a 3 kg infant, the CarpeDiem^TM^ machine was found inadequate to decrease serum ammonia in <4 h. Increasing body weight (4 kg) resulted in longer time intervals to reach the target.

**Figure 3:**
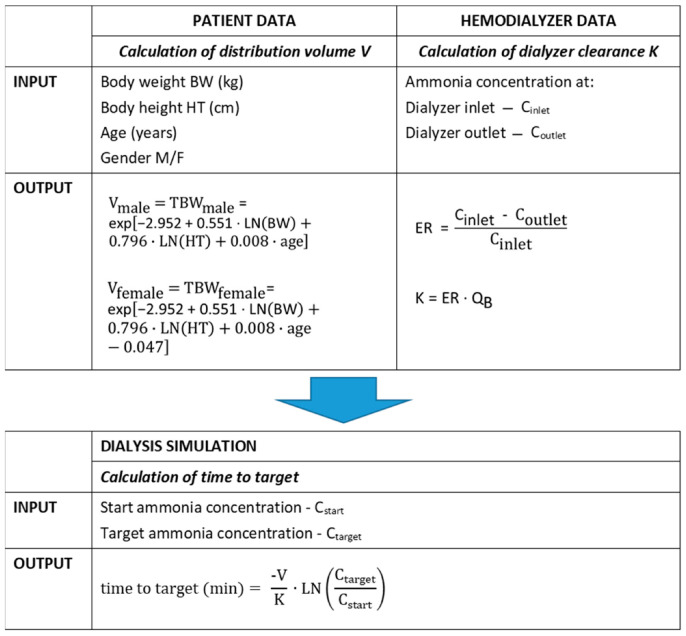
Algorithm to predict ammonia concentration decline.

Figure 3 presents the algorithm to calculate the time needed to decrease the ammonia concentration from a predialysis start concentration C_start_ to a chosen target concentration C_target_, accounting for the patient’s characteristics (i.e., body weight, height, age and gender) and dialysis setup characteristics (i.e., dialyzer clearance). By implementing these formulae in a simple spreadsheet, medical staff can draw an institution-specific flowchart for patient-tailored treatment of hyperammonemia.

**Figure 4:**
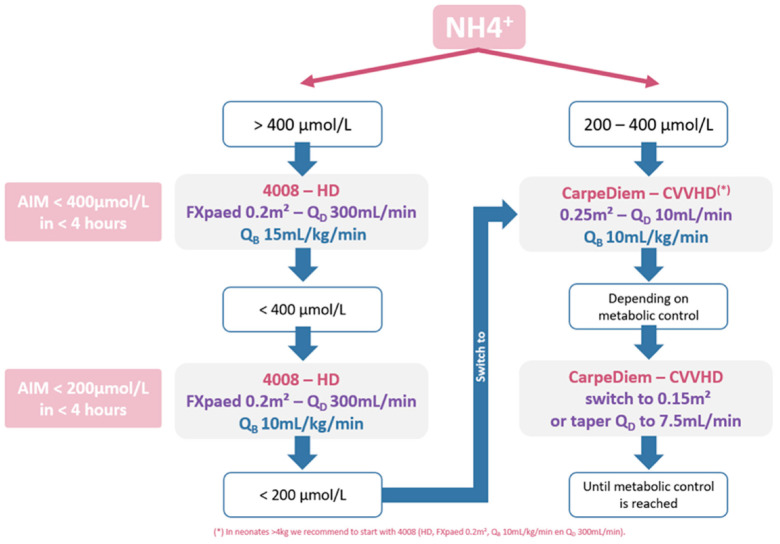
Flow chart of the proposed protocol.

Figure 4 shows an example of a clinical protocol for an infant of 2–4 kg, derived from the presented algorithm and accounting for our institution-specific procedures, treatment goals and infrastructure. To reduce the ammonia concentration to below 400 µmol/L as quickly as possible, preferably within 4 h, we decided upon starting KRT with the Fresenius 4008/FXPaed dialyzer (i.e., extraction ratio of 45%) for ammonia start concentrations higher than 400 µmol/L (Figure 4). Starting with a blood flow Q_B_ of 15 mL/kg/min, Q_B_ can be lowered to 10 mL/kg/min once the ammonia concentration drops below 400 µmol/L. For ammonia concentration below 200 µmol/L, the CarpeDiem^TM^ dialysis machine can be used with the 0.25 m^2^ dialyzer and Q_B_ 10 mL/kg/min. To avoid any rebound in between the dialysis sessions, the off-dialysis time should be limited as much as possible. On the basis of the plasma ammonia concentration and the established metabolic control, dialysis efficiency can be further decreased either by using the smaller dialyzer with the CarpeDiem^TM^ (i.e., 0.15 m^2^), or by lowering the dialysate flow from 10 to 7.5 mL/kg/min. However, in the case dialysis efficiency seems to become inadequate, a step back should be taken in the proposed flowchart (Figure 4). In cases where the infant weighs more than 4kg and has a start ammonia concentration between 200 and 400 µmol/mL, the 4008/FXPaed dialysis setup was chosen to reach ammonia levels <200 µmol/mL in less than 4 h.

## 3. Discussion

Acute hyperammonemia is a major cause of mortality among neonates with inborn errors of metabolism. Current guidelines on management of acute neonatal hyperammonemia are based on scarce evidence, and ammonia kinetics in the patient–dialysis setup is poorly understood. The main contribution of this study is the development of a patient-based ammonia kinetic model that results in a bedside-available algorithm to optimize KRT prescription of neonates with acute hyperammonemia, and to allow development of practical clinical protocol, while taking into account center-specific resources. 

The present simulations clearly show that adequate dialysis is needed to obtain a rapid decline in ammonia concentration, a key determinant of neurological outcome [19]. To obtain a high dialysis clearance of ammonia, which is, like urea, mainly diffusing, high blood and dialysate flows and dialyzer membrane surface area are the most important parameters for success. In the neonatal setting, however, blood flow might be compromised by the lack of appropriate vascular access [20] and dialyzer dimensions should be kept small to limit the extracorporeal volume [21]. Intermittent HD may lead to saw-tooth kinetics of ammonia levels, and to fluctuations of serum electrolytes, pH, body temperature and fluid balances. Hence, rapid fluid shifts, related to the treatment interruptions, have previously been found to even worsen cerebral edema [22]. In comparison to intermittent HD, CRRT is better tolerated in neonates and an improved 2-years survival has been demonstrated [23].

As an answer to the absence of licensed and adapted hemodialysis machines for infants on the market, the CarpeDiem^TM^ (Cardiac Renal Pediatric Dialysis Emergency Machine) (Medtronic, Minneapolis, MN, USA) has been developed to allow safe application of CRRT in infants [24]. While the small extracorporeal volume, using a miniaturized device, avoids the risk of hemodynamic derangements, the present study shows that efficiency of this dialysis machine is inadequate to establish a rapid initial decline in ammonia concentration. In addition to the pharmacological management with ammonia scavengers, the standard of care for neonates presenting with severe hyperammonemia should therefore be high-dose CRRT [25,26]. Our present findings are in agreement with Spinale et al. [26], who state that high-dose dialysis should be considered when ammonia levels are 400 µmol/mL or higher, and that blood flows should be 30–50 mL/min until the ammonia level has been reduced to at least 100–200 µmol/L. Such high-dose CRRT requires the use of dialysis machines comparable to the ones used in the adult dialysis setting, and which are, apart from the pediatric dialyzer, not designed to be used in a neonatal setting. In such cases, priming of the large extracorporeal circuit is inevitable.

Although the present study provides the clinician with a tool to predict the evolution of ammonium levels, with treatment depending on the available dialysis machines, it is important to understand the underlying fundaments of the presented algorithm and its limitations. From the intradialytic exponential time–concentration profiles in the four neonates with hyperammonemia, it was assumed that ammonia is distributed in a single volume. While this assumption holds true for the isolated effect of dialysis, the exponential curves are actually the result of the effect of hemodialysis plus ongoing ammonia generation, as well as pharmacological metabolic correction. The interplay of these phenomena might explain the intra- and post-dialytic ammonia rebound that has been reported previously [27], and thus is less likely to be related to the multicompartmental behavior of ammonia. Deriving dialyzer clearance from this exponential curve, as performed in the present analysis, can however still result in over- or underestimation of clearance. The algorithm therefore proposes to derive dialyzer clearance from direct concentration measurements at the dialyzer inlet and outlet. Additionally, the distribution volume of ammonia was assumed to be equal to the patient’s total body water volume. While this assumption fits in the story of urea, further validation of the ammonia model should be performed to confirm it. Even if true, it is important to be aware of the sensitivity of Wells’ formula [24], including weight, height, age and gender to calculate total body water. After all, the relative water volume changes rapidly the first days after birth and fluid overload is often present in critically ill patients. However, apart from all these interplaying factors and from the limited number of included neonates (i.e., four), consistent kinetic data could be obtained. Finally, in the present analysis, generation was derived from the interdialytic concentration increase, assuming a steady state in the patient, and showing a resultant generation rate not influencing dialyzer extraction. A more accurate calculation of generation, based on measured ammonia concentrations in urine and waste dialysate, should help to validate the present model, to follow-up on any influence of generation on dialyzer extraction and to simulate ammonia concentrations and, with it, a patient’s clinical status, in the interdialytic periods. In general, it should be highlighted that outcome data are very limited and that it remains difficult to elucidate the individual effect of the KRT in addition to other therapies such as protein-free diets and metabolic correction with amino acid therapies.

Although preliminary, the present kinetic model resulted in a useful algorithm as a tool to conceptualize a center-specific protocol to treat acute neonatal hyperammonemia fitting the patient’s characteristics. This tool might be an important link in the decision-making process, taking into account the availability of dialysis equipment and/or staff, treatment goals and overall condition of the patient, the trend in serum ammonia levels, the response to nitrogen-scavenger therapy and the age and body characteristics of the infant.

## 4. Materials and Methods

### 4.1. Patients, Dialyses and Blood Sampling

This study included all patients admitted in 2019–2020 at the pediatric intensive care unit of the Ghent University Hospital, Belgium, who were treated for neonatal hyperammonemia with hemodialysis. For this treatment, one of three dialysis options could be chosen: the 4008 dialysis machine, the FXPaed dialyzer (0.2 m^2^, Fresenius Medical Care, Bad Homburg, Germany), or the CarpeDiem^TM^ dialysis machine (0.15 m^2^ and 0.25 m^2^ dialyzer) (Medtronic, MN, USA). The characteristics of both machines and dialyzers are given in Table 4.

For clinical follow-up during the treatment, blood sampling was performed in the intra- and interdialytic period, targeting hourly sampling. All blood samples were centrifuged, and plasma was analyzed for ammonia using enzymatic determination with the glutamate dehydrogenase method in the Routine Laboratory of the Ghent University Hospital.

### 4.2. Calibration of the Kinetic Model

**Figure 5:**
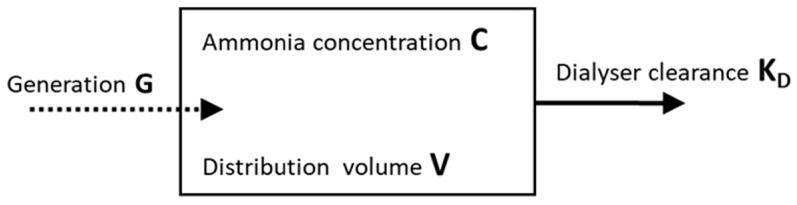
One-compartmental kinetic model.

The intradialytic ammonia plasma concentrations were used to model ammonia transport during hemodialysis based on a one-compartmental kinetic model (Figure 5). Herewith, the compartmental volume resembles the distribution volume V of ammonia, which was considered to be total body water, calculated by the formula of Wells et al. [20] using body weight, height, age and gender. This compartmental volume is mathematically described, having a homogeneous ammonia concentration and different inputs (i.e., generation) and outputs (dialyzer clearance). Dialyzer clearance K (mL/min) was calculated from the initial ammonia concentration decline in a single compartmental kinetic model, as described by:

C = C_0_∙exp((-K∙t)/V) (1)

with C_0_ (µmol/L) representing the ammonia concentration at the dialysis start, t (min) representing the time point during dialysis and V (mL) representing the total distribution volume. The ultrafiltration flow (i.e., patient’s weight loss) was negligible and not further considered. Extraction ratio ER (%) was derived from dialyzer clearance K_D_, accounting for dialysis blood flow Q_B_, (i.e., ER = K/Q_B_). Generation (µmol/min), i.e., the result of the ammonia production and the medical management such as nitrogen-scavenging agents, was derived from the ammonia concentration increase during an interdialytic steady state period (Figure 1):

(2) $$G=\left(C_{\text{end}}-{\text{C}}_{\text{start}}\right)\cdot \frac{V}{\Delta t_{\text{inter}}}$$

with Cstart and Cend (µmol/L) being the ammonia concentrations at the start and end of the interdialytic period, Δt (min) being the time interval of the interdialytic period and V (mL) being the total distribution volume.

### 4.3. Kinetics Simulations

The calibrated single compartmental model was further used to simulate plasma ammonia concentration profiles in different dialysis strategies during a period of 24 h. Simulations were performed in infants of 3 and 4 kg, height 50 cm, age 3 days and male, resulting in a total body water of 72% (BW 3 kg) and 63% (BW 4kg) as calculated according to Wells et al. [20].

Ammonia concentration declines were simulated for different start concentrations, i.e., 3000, 1500, 800, 400 and 200 µmol/L. From the derived extraction ratios of the corresponding dialysis setups, dialyzer clearances were calculated for a blood flow of 30 mL/min (all dialysis setups), and for 50 mL/min (4008/FXPaed setup), covering feasible blood flow rates in this patient population. Dialysate flow was 300 (4008/FXPaed) and 10 mL/min (CarpeDiem^TM^).

Starting from an infant’s body characteristics (weight and length) and its ammonia start concentration, the simulated concentration decline, per dialysis setup, provided the time needed to reach the thresholds 400 and 200 µmol/L, respectively. Comparing these time intervals allowed us to propose a flowchart, indicating which dialysis machine and which operating conditions to use in each particular case.

### 4.4. Optimized and Personalized Dialysis Protocol

Based on pre-defined criteria and the results of the kinetic study, we defined a flow chart to manage acute neonatal hyperammonemia. As no international guideline has proposed a validated ammonia threshold nor an acceptable timeframe for ammonia to drop, our local multidisciplinary team defined in consensus criteria for a successful treatment of acute neonate hyperammonemia while taking into account the resources present in our unit and the established toxicity of ammonia levels >200 µmol/L. The defined treatment goals we aimed for in our local flow chart were: (i) decrease ammonia level <400 µmol/L within 4 h after initiation of dialysis, subsequently (ii) decrease serum ammonia to <200 µmol/L in the next 4 h and (iii) minimize the rebound of ammonia concentrations (>200 µmol/L) after the hemodialysis stop.

In order to allow medical staff all around the world to benefit from the present kinetic findings, the algorithm is provided to calculate the dialysis time needed to reach a predefined desired threshold.

## Figures and Tables

**Table 1:** Patient’s characteristics.

	Patient 1	Patient 2	Patient 3	Patient 4
**Patient characteristics at start hemodialysis**				
Age (days)	3	3	3	5
Sex (F/M)	M	M	M	F
Body weight (kg)	3.15	3.82	3.02	2.96
Body length (cm)	48	54	52	48.5
Calculated TBW (L)	2.14	2.62	2.23	2.04
Diagnosed IEM	OTC	OTC	OTC	MMA
Serum ammonium (µmol/L)	1377	729	3017	709
Time from diagnosis to start hemodialysis	06 h 10 min	11 h 40 min	15 h 20 min	6 h 13 min
**Dialysis prescription**				
Vascular access	6.5 Fr dL IJV	6.5 Fr dL IJV	6.5 Fr dL IJV	6.5 Fr dL IJV
Fresenius 4008 (n = number of sessions) (priming: PC or S)	1 (PC)	1 (PC) + 1 (S)	1 (PC)	1 (PC)
CarpeDiem CD015 (n = number of sessions) (priming: PC or S)	2 (S)	0	0	0
CarpeDiem CD025 (n = number of sessions) (priming: PC or S)	1 (S)	1 (S) + 2 (PC)	0	0
Anticoagulation	Heparin	Heparin	Heparin	Heparin
**Therapy and outcome**				
Duration medical management * prior to hemodialysis initiation	01 h 23 min	09 h 15 min	13 h 15 min	1 h 30 min
Time between arrival at our center and hemodialysis initiation	01 h 23 min	00 h 57 min	01 h 14 min	01 h 30 min
Time from HD initiation to serum ammonia <400 µmol/L	04 h 06 min	01 h 44 min	06 h 43 min	2 h 17 min
Time from diagnosis to reaching serum ammonia <400 µmol/L	10 h 16 min	13 h 24 min	22 h 03 min	8 h 30 min
Survival	Yes	Yes	Yes	No

IEM: inborn error of metabolism, OTC: ornithine transcarbamylase deficiency, MMA: methylmalonic acidemia, dL: double lumen, IJV: internal jugular vein, F: female, M: male, L: liter. (*): sodium benzoate and sodium phenylacetate, (PC): 50% packed cells and 50% albumin solution, (S): saline.

**Table 2:** Time needed for the ammonia concentration to reach the threshold concentration in a male infant of 3 kg, for different dialysis setups and different blood flows.

Start Concentration		3000 µmol/L		1500 µmol/L		800 µmol/L		400 µmol/L		200 µmol/L	
Setup	Threshold	Q_B_ 30	Q_B_ 50	Q_B_ 30	Q_B_ 50	Q_B_ 30	Q_B_ 50	Q_B_ 30	Q_B_ 50	Q_B_ 30	Q_B_ 50
4008	400	322	193	211	126	111	66	-	-	-	-
	200	433	259	321	193	221	133	111	67	-	-
	100	546	326	431	259	330	199	221	133	110	65
CD025	400	965	-	632	-	332	-	-	-	-	-
	200	>>	-	965	-	662	-	332	-	-	-
	100	>>	-	>>	-	995	-	665	-	330	200
CD015	400	1112	-	730	-	382	-	-	-	-	-
	200	>>	-	1112	-	765	-	382	-	-	-
	100	>>	-	>>	-	1150	-	765	-	385	230

Q_B_: blood flow (mL/min); CD: CarpeDiem; >>: more than 24 h.

**Table 3:** Time needed for the ammonia concentration to reach the threshold concentration in a male infant of 4 kg, for different dialysis setups and different blood flows.

Start Concentration		3000 µmol/L		1500 µmol/L		800 µmol/L		400 µmol/L		200 µmol/L	
Setup	Threshold	Q_B_ 30	Q_B_ 50	Q_B_ 30	Q_B_ 50	Q_B_ 30	Q_B_ 50	Q_B_ 30	Q_B_ 50	Q_B_ 30	Q_B_ 50
4008	400	471	283	309	186	162	97	-	-	-	-
	200	632	380	471	282	324	194	162	97	-	-
	100	795	476	632	380	485	291	325	195	163	97
CD025	400	1414	-	926	-	486	-	-	-	-	-
	200	>>	-	1413	-	972	-	485	-	-	-
	100	>>	-	>>	-	>>	-	970	-	485	-
CD015	400	>>	-	1070	-	560	-	-	-	-	-
	200	>>	-	>>	-	1120	-	560	-	-	-
	100	>>	-	>>	-	1680	-	1120	-	560	-

Q_B_: blood flow (mL/min); CD: CarpeDiem; >>: more than 24h.

**Table 4:** Main characteristics of the applied dialysis machines, dialyzers and operating parameters.

Company	Fresenius Medical Care	Medtronic
**Dialysis machine**	**4008**	**CarpeDiem**
Blood flow (mL/min)	30–100	5–50
Dialysate flow (mL/min	300–800	10
**Dialyzer**	**FXPaed**	**HCD 015/HCD 025**
Surface area (m^2^)	0.20	0.17/0.29
Fiber material	Helixone^®^	Polyethersulfon
Priming volume (mL)	18	11/20
Fiber diameter (µm)	220	200
Membrane thickness (µm)	35	30
Total priming volume (mL)	53	32/41

## Data Availability

The data presented in this study are available on request from the corresponding author. The data are not publicly available due to privacy restrictions.
